# Dentition as Affected by Rickets

**Published:** 1889-10

**Authors:** 


ARTICLE V.

### DENTITION AS AFFECTED BY RICKETS.

If rickets prevail to any extent in American communities, it must exercise a very important influence in all diseases of childhood, and must play an exceedingly important role in infant-mortality. As dentition is almost always retarded and irregular in rickets, I may be permitted to dwell at some length upon its pathology. It is a stormy epoch with children. Although a physiological process, which should be regular in its course and without danger to life, nevertheless all authors agree that any illness occurring during teething is aggravated in type and dangerous and protracted in course. Convulsions, diarrhœa, bonchitis are common in these critical months, and the mortality among children from six to twenty-four months of age, in Buffalo, is 1,500 yearly. I even find dentition ranked as a cause of death, sixteen deaths from dentition being returned. Yet teething goes smoothly on in healthy children, the central incisors appearing at the seventh month, the lateral at the ninth, the first molars at the twelfth, the canine at the eighteenth and the second molars at the twenty-fourth month. Any essential deviation from this order is a variation from the standard of health, and its cause should be sought for and explained. Delayed dentition is not due to all causes which enfeeble a child, for in infants suffering from diarrhœa or tuberculosis the eruption of the teeth is regular, and the teeth are often well formed and strong. Among the laity, many maladies of childhood are referred to the malignant influence of teething, and mothers ask their physicians why their children are so fretful and sickly, and why the teeth do not appear at the appointed time. The maternal anxieties are usually quieted with soothing assurances that all will be well in time, but the most common cause of delayed dentition is rickets. A rickety child may have no teeth even as late as the second year, or the eruption of teeth may progress regularly until the incisor teeth are through, and the child becoming rickety, dentition may be arrested for months. These rachitic teeth are very prone to decay, and physicians have observed how very bad the teeth are among the children of the city poor. No physical examination of a sick child is complete without a careful inquiry into the condition of the teeth. If dentition be late or irregular, and if the child be harassed by digestive troubles, or obstinate pulmonary catarrh, the underlying cause will almost invariably be found to be rickets. I will quote again from Sir William Jenner upon this subject:

"If a child pass over the ninth month without teeth, you should carefully inquire for the cause. It may be that an acute illness has retarded dentition. It may be, but this is very rare, that there is some condition of the jaw which interferes with the advance of the teeth ; it may be, and this is infinitely the more common cause of late dentition, that the child is rickety. Fail not, then, when called to a child in whom the teeth are late in appearing, to look if it be rickety, for if you fail to look for rickets you will most likely attribute to the irritation of teething symptoms which are the consequence of the rickety diathesis, the late dentition of rickets being in itself a symptom of the general disorder. The rickety deformities may be very trifling, and yet the teeth be considerably retarded in their development."—*Medical Record.*

